# Changing diagnostic criteria and their impact on metabolic syndrome: a systematic review

**DOI:** 10.3389/fendo.2026.1976380

**Published:** 2026-10-20

**Authors:** Ahmad A. Obeidat, Dalia M. Abu Al-Haijaa, Razan J. Rahahleh, Mousa N. Ahmad, Sarah Jaradat, Najla Al Nassar, Nermin A. Khamis, Firas S. Azzeh

**Affiliations:** 1 Human Nutrition and Dietetics, Department of Nutrition and Food Technology, The University of Jordan, Amman, Jordan; 2 Department of Therapeutic Nutrition and Dietetics, College of Applied Medical Sciences, Amman Arab University, Amman, Jordan; 3 Department of Diet Therapy Technology and Dietetics, Faculty of Allied Medical Sciences, Zarqa University, Al-Zarqa, Jordan; 4 Human Nutrition and Dietetics, College of Health Sciences, Abu-Dhabi Universityi, Abu-Dhab, United Arab Emirates; 5 Head of Preparatory Year Program, Batterjee Medical College, Madinah, Saudi Arabia

**Keywords:** diagnostic concordance, diagnostic criteria, ethnicity-specific thresholds, metabolic syndrome, waist circumference

## Abstract

**Introduction:**

The prevalence of metabolic syndrome (MetS) is difficult to define because there is no universal consensus on the definition of MetS and similarities and differences in definitions of the components and the architecture of the definitions can significantly affect the prevalence and individual classification. The aim of this systematic review was to assess the impact of alternative definitions of MetS on prevalence, diagnostic agreement, reclassification, metabolic components and ethnicity-specific classification.

**Methods:**

A systematic review was carried out following the PRISMA 2020 guidelines. Studies met inclusion criteria if they included a comparison of 2 or more MetS diagnostic definitions in the same adult population. Information was gathered about study characteristics, diagnostic frameworks, thresholds for the components of MetS, prevalence of MetS, agreement between the two diagnostic methods, reclassification, and regional or ethnic specific criteria. A descriptive synthesis of finding was completed because of the significant degree of population and diagnostic heterogeneity.

**Results:**

Nine studies were included in the evidence synthesis. The prevalence of MetS was highly heterogeneous when the same population was assessed using different diagnostic criteria. The prevalence rates in Finnish adults ranged from 17.7% (WHO criteria) to 43.0% (JIS criteria), and the prevalence rates in the nationwide Iranian study ranged from 32.0% (IDF criteria) to 47.6% (JIS criteria). There was high diagnostic concordance between structurally similar definitions such as between IDF versus JIS in Finland (κ = 0.97) while lower levels of concordance were found between comparisons using WHO criteria. The most frequently reported factors associated with diagnostic discordance were central obesity and waist circumference. In addition, there were significant variations in prevalence of MetS depending on the region-specific adjustment of waist-circumference thresholds. Furthermore, the prevalence of MetS varied significantly by region-specific adjustment of waist circumference thresholds.

**Discussion:**

The prevalence of MetS from one diagnostic framework to another may not be directly comparable since it is highly definition-dependent. Waist thresholds may vary by ethnicity and this can be a significant factor in classification. Further research should include precise diagnostic criteria and thresholds, cross-classification data and validation of alternative definitions with clinically relevant outcomes.

## 1 Introduction

Metabolic syndrome (MetS) is a cluster of clinically important cardiometabolic abnormalities, especially central obesity, hypertension, elevated triglycerides, low high density lipoprotein cholesterol (HDL-C) and impaired glucose regulation (1). MetS is a construct of great importance in population level risk assessment and preventive medicine as the individuals with this clustering are at higher risk of developing type 2 diabetes and cardiovascular disease (2). The concept of MetS is not a disease entity with a universal definition but rather a collection of metabolic risk factors which can manifest themselves differently in various populations (3). In addition, recent evidence suggests that the levels of cardiovascular risk associated with different combinations of MetS components may differ, suggesting that the clinical significance of MetS phenotypes may not be the same (4). With the lack of a single, consistent definition, a number of diagnostic algorithms are in use, such as the World Health Organization (WHO), the National Cholesterol Education Program Adult Treatment Panel III (NCEP-ATP III), Joint Interim Statement (JIS), American Heart Association/National Heart, Lung, and Blood Institute (AHA/NHLBI), and International Diabetes Federation (IDF) criteria (5). They vary with respect to the architecture of the underlying diagnosis, whether insulin resistance is included or not, whether the presence of central obesity is mandated, whether two or three abnormalities are necessary, and how the individual abnormalities are weighted, for example, in some, hypertriglyceridemia and low HDL are given equal weight, while in others a combination of high blood pressure, raised HDL, and hyperglycemia carries more weight (6). The variation is directly relevant to epidemiological surveillance and/or cross-population comparisons. If populations have been diagnosed as having MetS by different criteria, a difference in the prevalence of MetS does not necessarily mean a corresponding difference in the underlying cardiometabolic risk (7). Evidences from recent studies in high-income countries indicate significant racial differences in the prevalence of MetS even under the same NCEP-ATP III definitions, suggesting that the population characteristic and diagnostic criteria play a combined role (8). Furthermore, recent Iranian data still shows high variations between prevalence rates based on different definitions, as JIS tends to overestimate compared to IDF and NCEP-ATP III criteria (9). Central obesity is one area of diagnostic heterogeneity that deserves special mention. Waist circumference is affected by ethnic group, gender, age and body-fat distribution, which could mean that a single threshold may be used to classify metabolic risk differently across ethnic groups. This is especially important in Asian populations, where metabolic abnormalities may develop at lower levels of adiposity. Iranian evidence has shown that population-specific levels of WC can have a significant impact on MetS diagnosis, and new evidence from multiple countries has revealed a significant ethnic variation in MetS prevalence (8). The use of ethnic-specific waist thresholds in the nationwide Iranian study, which resulted in a substantial difference in population-level reclassification (from 43.5% with the international IDF criteria to 32.0% with the regional IDF criteria), shows how a single diagnostic component can be different, leading to significant reclassification among a population (10). Some studies have compared other definition of MetS, but the evidence is not conclusive. Past studies have frequently addressed prevalence in individual populations, and less emphasis has been placed on the amount of reclassification within populations, on pairwise agreement in diagnostic categories, on the within-population sources of disagreement, or on the role of ethnicity-specific thresholds (11, 12). In addition, high agreement between two definitions is not necessarily diagnostic superiority as the measurement of agreement is between definitions and not with a biological or clinical gold standard. This distinction is especially relevant for the interpretation of κ statistics and in the choice between the two definitions as a clinical risk predictor (13). Thus, a meta-synthesis of studies that directly compare several diagnostic definitions in the same populations is required. This approach can separate out variation due to differences in diagnostic architecture from variation due to differences between populations and make a more informative evaluation of diagnostic heterogeneity. The aim of the present systematic review was thus to assess the impact of alternative definitions on the prevalence and classification of MetS in adults, particularly the impact of the definition on prevalence, the concordance and reclassification of MetS, the role of MetS components in creating discordance, and the influence of regional or ethnicity-specific thresholds for diagnosis. By integrating these comparative findings, this review seeks to clarify the extent to which diagnostic methodology influences reported MetS burden and to identify priorities for improving diagnostic consistency, epidemiological comparability, and future outcome-based validation of MetS definitions.

## 2 Materials and methods

### 2.1 Study design and reporting framework

The purpose of this systematic review was to assess the impact of varying diagnostic definitions on the prevalence rate of metabolic syndrome (MetS) in adults, and to see how often these definitions resulted in different classifications of MetS, metabolic syndrome components, the reclassification of metabolic syndrome, diagnostic concordance, and ethnic-specific diagnostic thresholds. The review was conducted and written following the Preferred Reporting Items for Systematic Reviews and Meta-Analyses (PRISMA) 2020 statement and the PRISMA 2020 checklist (14). The review was designed as a comparative diagnostic-definition systematic review, and not as a traditional prevalence meta-analysis. Therefore, the key unit of comparison was the population in which two or more MetS definitions were applied to the same individuals to minimize the confounding that can occur if prevalence data from different populations are compared.

### 2.2 Review question and objectives

The main review question was: What effect does the use of various definitions of the diagnosis have on the classification and prevalence of adults with metabolic syndrome? The primary purpose of the review was to compare prevalence of MetS determined by different definitions in the same populations, to measure diagnostic agreement and disagreement between definitions, to determine metabolic measures that differed with each classification, and to determine if differences in classification varied by population characteristics (age, sex, ethnicity, and clinical setting). The conceptual framework for the review included a discussion of diagnostic definitions and thresholds used to make the diagnosis, subsequent metabolic classification, prevalence, reclassification, and diagnostic concordance and discordance. The evidence-extraction protocol thus focused on studies that used two or more diagnostic definitions in the same study population (15, 16).

### 2.3 Eligibility criteria

Studies were deemed to meet the criteria if they involved adult subjects (18 years and older) and compared two or more established diagnostic criteria for MetS within the same population. Eligible were community based, population based, epidemiological, nationally representative, and suitable clinical adult populations. Studies in which only children or adolescents were studied, animal studies, and experimental investigations were not included. The various diagnostic frameworks included were the World Health Organization (WHO), European Group for the Study of Insulin Resistance (EGIR), National Cholesterol Education Program Adult Treatment Panel III (NCEP-ATP III), American Association of Clinical Endocrinology (AACE), International Diabetes Federation (IDF), American Heart Association/National Heart, Lung, and Blood Institute (AHA/NHLBI), Joint Interim Statement (JIS), harmonized criteria and other recognized or explicitly described modified definitions. As long as the studies did not only apply a modified or regional definition, the precise diagnostic architecture and any changes to the applied criteria were extracted and taken into account when synthesizing the studies. Observational studies such as cross sectional, population-based studies, cohort derived studies, and other types of appropriate observational studies were eligible if they included reports of prevalence, classification, diagnostic agreement, reclassification, or comparative information relevant to the review question. Reviews, systematic reviews, meta-analyses, editorials/commentaries and studies that assessed only one MetS definition were excluded. Other studies were also excluded if the diagnostic criteria were unclear or if there was not enough information to classify or determine the prevalence result of the study. These eligibility principles were aligned with the comparative evidence framework that had been set up for the review (16–18).

### 2.4 Information sources

PubMed/MEDLINE, Scopus, Web of Science Core Collection, and Embase were searched from database inception to 31 July 2026. These four databases were the sources represented in the PRISMA flow diagram. Backward reference-list screening and forward citation searching were also undertaken to identify additional eligible reports. No other bibliographic database contributed records to the final search accounting.

### 2.5 Search strategy

The search combined controlled vocabulary and free-text terms for MetS, diagnostic definitions, named diagnostic frameworks, and comparative classification outcomes. The PubMed/MEDLINE strategy was: (“Metabolic Syndrome”[Mesh] OR “metabolic syndrome”[tiab] OR “metabolic syndrome X”[tiab] OR “insulin resistance syndrome”[tiab] OR “cardiometabolic syndrome”[tiab] OR “dysmetabolic syndrome”[tiab]) AND (“Diagnosis”[Mesh] OR diagnos*[tiab] OR defin*[tiab] OR criteri*[tiab] OR classification[tiab]) AND (WHO[tiab] OR EGIR[tiab] OR “NCEP-ATP III”[tiab] OR “ATP III”[tiab] OR IDF[tiab] OR AACE[tiab] OR “AHA/NHLBI”[tiab] OR “Joint Interim Statement”[tiab] OR harmoniz*[tiab]) AND (“Prevalence”[Mesh] OR prevalence[tiab] OR agreement[tiab] OR concordance[tiab] OR discordance[tiab] OR kappa[tiab] OR reclassification[tiab] OR compar*[tiab]). Relevant MeSH terms were Metabolic Syndrome, Diagnosis, and Prevalence; Waist Circumference and Ethnicity were included as supplementary controlled-vocabulary/free-text concepts where supported by the database. The syntax was adapted for Scopus, Web of Science, and Embase using their respective field tags and thesauri. No language, publication-date, or publication-type restrictions were applied at the database-search stage. Human adults (>=18 years) and eligible study designs were enforced during screening rather than by database filters. Searches were last updated on 31 July 2026.

### 2.6 Study selection

After duplicate removal, titles and abstracts were screened against the prespecified eligibility criteria, followed by full-text assessment. Eligibility at full text required an adult population, application of at least two established MetS definitions to the same participants, and reporting of a relevant comparative outcome. Reasons for exclusion included an ineligible population, application of only one definition, insufficient description of diagnostic criteria, an ineligible study design, duplicate or overlapping populations, and inadequate comparative data. Sixty-six reports were assessed in full text; 54 were excluded at this stage, leaving 12 eligible candidate reports. Three reports were subsequently excluded because they represented overlapping cohorts or did not provide independently verifiable comparative data, leaving nine studies for qualitative synthesis (**Figure 1**).

**Figure 1:**
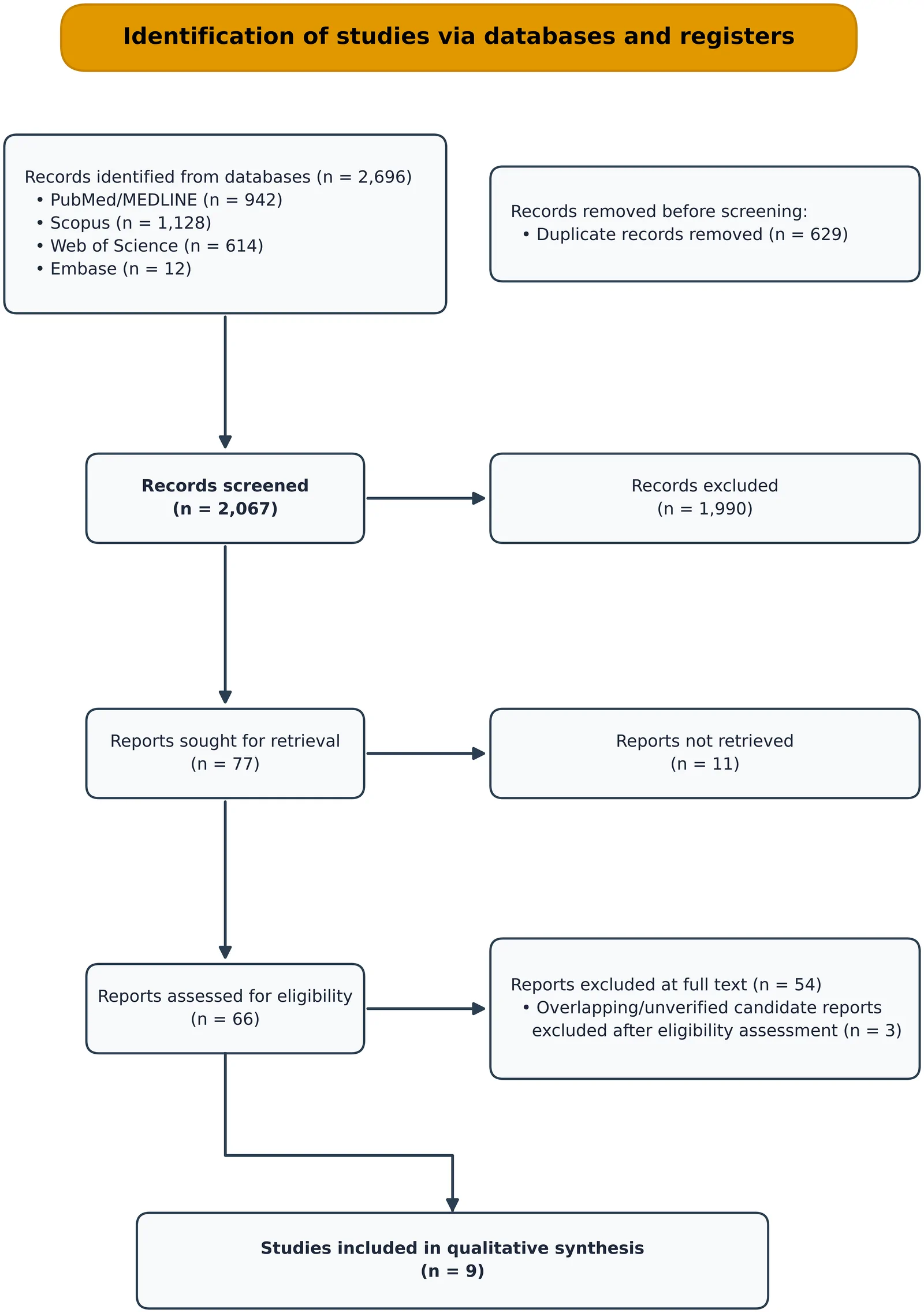
PRISMA 2020 flow diagram showing the identification, screening, eligibility assessment, and inclusion of studies in the systematic review.

### 2.7 Duplicate publications and cohort overlap

Bibliographic features such as title, authorship, publication year, journal, DOI and PMID were used to determine potential duplicate publications. The potential for overlapping populations was then assessed according to the following criteria: study population, study period, sample size, geographical distribution, and characteristics of the cohorts being recruited. If more than one publication was found for the same population, these publications were checked to see if they were duplicate analyses or if they were unique diagnostic comparisons or if they were unique information on subgroups. When significant overlap was established, the most informative report was included in the main synthesis, and other reports were kept only when they provided other relevant information for the review objectives (19, 20).

### 2.8 Data extraction

All studies were surveyed using a uniform data extraction form. Extracted variables were bibliographic characteristics, country and geographical setting, study design, population characteristics, sample size, age, sex, ethnicity, clinical status, diagnostic definition, thresholds of metabolic abnormalities used in components, required metabolic abnormalities, prevalence of MetS, prevalence of individual metabolic components, and measures of diagnostic agreement/reclassification. WHO, EGIR, NCEP-ATP III, AACE/ACE, IDF, AHA/NHLBI, JIS/harmonized, and regional/ethnicity-specific definitions have been reported separately to prevent the confusion of using diagnostically different frameworks interchangeably. Cohen’s κ, percentage agreement, sensitivity, specificity, predictive values and cross-classification data were obtained where available (15, 16, 20).

### 2.9 Diagnostic-definition harmonization

Diagnostic definitions were assessed for their underlying diagnostic architecture and not just published name. Insulin-resistance requirements, number of abnormalities required for MetS classification, individual component thresholds, the central obesity, and ethnicity-specific thresholds of waist-circumference were documented for each framework. This strategy enabled the differences in diagnostic architecture to be studied as they relate to differences in the definition of MetS, but not to change the original criteria reported in each study (16, 18, 19).

### 2.10 Data verification and missing-data management

Information obtained was cross-checked with the original publication before being added to the verified evidence set. For each sample size, participant characteristics, diagnostic criteria, component thresholds, prevalence of MetS, metabolic-component frequencies, reported measures of agreement were checked where possible in the original source. Data on variables not provided by the investigators were coded as NR (not reported) and data that could not be verified from the available original publication were not considered as verified evidence. There were no assumptions, secondary data, or unrelated studies to fill in missing values for numerical data (14, 21).

### 2.11 Risk-of-bias assessment

The Joanna Briggs Institute (JBI) critical appraisal tools that were specific to the design of each study included were used to assess the risk of bias. The methodological domains assessed were sampling frame, sampling method, sample size, participant description, identified population coverage, validity and reliability of outcome measurement, statistical analysis, and response rate, which are relevant to the study design of observational prevalence and analytical cross-sectional designs. Judgements were made at the individual domain level (yes, no, unclear, not applicable) and overall qualitative judgement. No numerical quality scores were computed as adding up the individual domains may mask differences in the type and significance of methodological shortcomings. The current appraisal should be considered a provisional appraisal as there is no indication that the evidence package was reviewed independently by another reviewer. This restriction must be clearly stated in the final text, not assumed as an independent duplicate appraisal which was not carried out (22).

### 2.12 Evidence synthesis

The differences in population characteristics, clinical setting, diagnostic architecture, waist circumference thresholds, ethnicity, age, sex, and measurement procedures among the included studies led to a descriptive synthesis of the evidence rather than pooling of data. Within the populations, in which various diagnostic criteria had been applied to the same individuals, differences due to the criteria of diagnosis were better distinguished from differences between populations of unrelated individuals in the principal comparison (16, 20, 21).

#### 2.12.1 Definition-specific prevalence

The prevalence rates of MetS within each study were retrieved and compared for each of the following diagnostic definitions. The maximum and minimum prevalence estimates were determined, and the absolute difference between definitions were determined when enough reported data were available. Prevalence differences were specifically reported by the original investigators, and were separated from calculated differences (14, 15, 19).

#### 2.12.2 Diagnostic concordance

Cohen’s κ and percentage agreement were calculated for diagnostic concordance as reported by the original investigators. Concordance was defined as the agreement between classification systems and not as a measure of the criterion validity or the superiority of any classification system, as no biological gold standard was available. If the participant-level cross-classification data was available, the percentages that were classified differently by the two competing definitions were also examined (14, 15, 19).

#### 2.12.3 Diagnostic reclassification

Within-study reclassification was assessed based on the percentage of individuals who had a different MetS diagnosis according to different definitions. Wherever the prevalence figures for two definitions were reported, the difference in absolute percentages was computed as a measure of the magnitude of the changes in prevalence due to the different definitions. Differences reported directly by the study investigators were kept separate from calculated differences to preserve transparency of the sources of each difference (14, 15, 19).

#### 2.12.4 Metabolic-component discordance

Across the definitions, the contribution of each metabolic component was explored to the discordance. Special focus was paid to central obesity or waist circumference, triglycerides, HDL-cholesterol, blood pressure, fasting glucose and insulin resistance. Central obesity is studied separately as the definition varies from one study to another (abdominal obesity is required for MetS definition or not) and the waist circumference thresholds used to define abdominal obesity (14, 15, 19).

#### 2.12.5 Ethnicity-specific classification

The regional and ethnicity specific modification of the definition of MetS was assessed independent of the international criteria. If adequate information was provided, the prevalence values based on international thresholds of waist circumference were compared directly with prevalence values based on regional and/or ethnicity-specific thresholds within the same population. This was employed to determine if there was a difference in population-specific thresholds for abdominal obesity in terms of classification of MetS or diagnostic concordance (14, 15, 19, 22).

#### 2.12.6 Subgroup analysis

Where sufficient information was available, findings were analyzed by geographical area, ethnicity, sex, age, population and clinical status. When possible, the clinical populations were analyzed separately from the general adult population as differences in baseline metabolic characteristics might affect the apparent impact of the definition of diabetes. Age- and sex-specific findings were aggregated descriptively since studies included used inconsistent age categories and different reporting procedures for age subgroups (14, 15, 19, 22).

### 2.13 Sensitivity analysis

Prespecified sensitivity analyses were conducted to determine if methodological differences between studies affected the main interpretation. When sufficient data were available, the synthesis was compared after excluding studies with moderate risk of bias, clinical samples instead of general population samples, substantially modified MetS diagnostic criteria, and/or incomplete verification of information. Populations potentially overlapping were also analyzed separately to check if the interpretation was affected by multiple reports from the same population. In the final manuscript, only sensitivity analyses performed should be reported as results. Where evidence is missing, a particular sensitivity analysis should be explicitly identified, and not shown as completed (23).

### 2.14 Assessment of reporting bias

The potential for reporting limitations was qualitatively evaluated by exploring the completeness of reporting of diagnostic definition, threshold for components, prevalence estimates, concordance measures, participant-level cross-classification, individual metabolic components, and relevant findings by subgroups of studies included. No formal statistical procedures for publication bias were planned as the review was not to estimate a single pooled effect and it was anticipated that the included studies would vary widely in both the diagnostic definition and population characteristics (14, 22, 24).

### 2.15 Quantitative synthesis

There was no pooled meta-analysis of the prevalence of MetS as the aim was to compare the alternative diagnostic definitions within populations not to derive a common prevalence across heterogeneous populations. The large differences in the diagnostic architecture, in the population characteristics, in ethnicity, age, and sex, in clinical status, and in component thresholds were thought to be incompatible with a clinically meaningful pooled prevalence estimate. The structured tabulation, within-study comparisons of prevalence, absolute differences in percentages, reported concordance measures, reclassification patterns, and interpretation at the component level were the key elements of the principal synthesis (14, 22, 24).

### 2.16 Statistical analysis

The characteristics of the studies and/or the results on the construction of the definition were summarized in a descriptive way. Estimates of prevalence were presented as percentages and differences in absolute percentages between alternative definitions were reported as differences when applicable. Where participants’ level information was available, Cohen’s κ and percentage agreement were extracted directly from the original studies instead of recalculated. This review was not intended to be a quantitative prevalence meta-analysis because no pooled prevalence, pooled effect estimate, I² statistic or conventional meta-analytic heterogeneity estimate was calculated (14, 19, 21, 22, 24).

## 3 Results

### 3.1 Study selection

The database searches identified 2,696 records: 942 from PubMed/MEDLINE, 1,128 from Scopus, 614 from Web of Science Core Collection, and 12 from Embase. After removal of 629 duplicates, 2,067 records underwent title and abstract screening; 1,990 were excluded. Of 77 reports sought for retrieval, 11 could not be retrieved, and 66 full-text reports were assessed for eligibility. Fifty-four full-text reports were excluded, leaving 12 eligible candidate reports. A further three reports were excluded after assessment of cohort overlap and independent data verification, resulting in nine studies in the qualitative synthesis. The included studies represented adult populations from Turkey, Seychelles, Iran, Finland, China, Ethiopia, and Pakistan (**Figure 1**).

### 3.2 Characteristics of the included studies

The nine included studies were published between 2007 and 2024 and included populations from Europe, the Middle East, Asia, and Africa. The most recent eligible publication was Adil et al., which reported a survey conducted in 2022 (17). Searches were updated through 31 July 2026; records published after 2024 were screened, but no 2025–2026 report met all eligibility criteria, particularly the requirement that at least two established MetS definitions be applied to the same adult population with extractable comparative outcomes. Sample sizes ranged from 314 Ethiopian adults with type 2 diabetes mellitus to 18,414 adults in a nationwide Iranian survey. The populations and settings were heterogeneous and were therefore retained for descriptive rather than pooled synthesis (**Table 1**).

**Table 1:** Characteristics of the verified studies included in the systematic review.

Study	Country	Population/setting	Sample size (N)	Diagnostic definitions evaluated	Main population characteristics
(32)	Turkey	Nondiabetic adults	1,568	WHO; EGIR; NCEP-ATP III; AACE; IDF	Adult Turkish population; prevalence estimates age- and sex-adjusted
(33)	Seychelles	Adult population	1,255	ATP III; WHO; IDF	Adult population from Seychelles; analyses also reported after exclusion of participants with diabetes
(34)	Iran	Tabari Cohort	NR	NCEP-ATP III; international IDF; Iranian-specific IDF	Adult Iranian cohort
(9)	Iran	Nationwide population-based survey	18,414	NCEP-ATP III; IDF; AHA/NHLBI; JIS; regional IDF; regional JIS	Nationwide Iranian adult population
(35)	Finland	Population-based adult population	5,687	WHO; NCEP-ATP III; IDF; JIS	Finnish population-based sample
(36)	China	Rural Chinese population	4,748	WHO; CDS; updated NCEP-ATP III; Asian-American; IDF; JIS	Rural Chinese adults; sex-specific prevalence reported
(37)	Ethiopia	Adults with type 2 diabetes mellitus	314	WHO; IDF; harmonized; NCEP-ATP III	Clinical population with type 2 diabetes mellitus
(17)	Pakistan	Apparently healthy adults, Karachi	1,065	NCEP-ATP III; IDF; modified NCEP-ATP III	Apparently healthy adult population
(38)	Iran	Iranian cohort, Shiraz	7,225	Modified WHO; NCEP-ATP III; AHA; IDF	Adult Iranian cohort; cardiovascular-risk-related analysis

NR, not reported or not verified in the current extraction record. The sample sizes and definition sets above follow the current verified evidence database.

### 3.3 Diagnostic definitions and diagnostic architecture

The constructs assessed differed significantly throughout the included studies. The most commonly represented frameworks were WHO, NCEP-ATP III, IDF, AHA/NHLBI and JIS or harmonized criteria, with fewer studies evaluating EGIR and AACE/ACE. The Turkish study was one of the largest historical comparisons, using WHO, EGIR, NCEP-ATP III, AACE and IDF criteria in the same population. Both the Seychelles study and the Finnish study used ATP III criteria, but the Finnish study also included WHO, NCEP-ATP III, IDF, and JIS criteria. A national study of Iranians was conducted to compare six diagnostic frameworks, some of which were the international and regional modifications of IDF and JIS criteria. There were variations in diagnostic criteria among the structural components of the diagnostic frameworks, such as the number of metabolic abnormalities needed for diagnosis, the inclusion of insulin resistance and the use of thresholds for waist circumference. Insulin-resistance related criteria were added to WHO and EGIR frameworks, while NCEP-ATP III, IDF, AHA/NHLBI and JIS/harmonized used combinations of routinely measured metabolic abnormalities. There were also differences in the thresholds for circumference of the waist, between definitions and between international and regional thresholds (**Table 2**).

**Table 2:** Diagnostic definitions, diagnostic architecture, and threshold characteristics evaluated across the included studies.

Diagnostic framework	Principal diagnostic architecture	Central obesity/waist circumference	Insulin resistance	Other principal components	Application in verified studies
WHO	MetS classification incorporating metabolic abnormalities with an insulin-resistance component	Included as a component; exact study-specific threshold varied	Central feature of framework	Glucose regulation, blood pressure, triglycerides, HDL-C and related abnormalities	(32, 33, 35–38)
EGIR	Insulin-resistance-centered framework with additional metabolic abnormalities	Included	Required/central component	Blood pressure, triglycerides, HDL-C, fasting glucose and obesity-related measures	(32)
NCEP-ATP III	Component-based definition requiring clustering of metabolic abnormalities	Waist circumference included as one component	Not obligatory	Triglycerides, HDL-C, blood pressure and glucose	(9, 32, 33, 37, 38)
AACE/ACE	Insulin-resistance/metabolic-risk-oriented framework	Included among metabolic-risk features	Important component	Glucose dysregulation and cardiometabolic risk factors	(32)
IDF	Component-based framework with central obesity as a key/obligatory feature in the classical formulation	Central obesity required in classical IDF; ethnicity-specific waist thresholds	Not obligatory	Raised triglycerides, reduced HDL-C, elevated blood pressure and glucose abnormality	(17, 37, 38)
AHA/NHLBI	Component-based framework broadly aligned with ATP III architecture	Included	Not obligatory	Triglycerides, HDL-C, blood pressure and glucose	(9, 38)
JIS/harmonized	Harmonized component-based framework; diagnosis based on clustering of metabolic abnormalities	Waist circumference included; population-specific cut-offs may be used	Not obligatory	Elevated triglycerides, reduced HDL-C, blood pressure and glucose	(9, 35–37)
Regional IDF	IDF architecture incorporating population-specific modification of abdominal-obesity threshold	Iranian-specific waist threshold	Not obligatory	Other IDF components retained	(9, 34)
Regional JIS	Harmonized/JIS architecture with regional modification	Regional waist threshold	Not obligatory	Other JIS components retained	(9)
Modified NCEP-ATP III	Modified ATP III architecture	Modified component/threshold structure as reported by the original study	Not obligatory	Standard ATP III metabolic components with study-specific modification	(17)
Modified WHO	Modified WHO framework as reported in the Iranian cohort	Study-specific modification	WHO-related insulin-resistance architecture retained as applicable	Study-specific metabolic components	(38)
CDS	Component-based Chinese diagnostic framework	Included	Study-specific	Cardiometabolic components	(36)
Asian-American criteria	Population-specific/ethnicity-related framework	Ethnicity-specific abdominal-obesity threshold	Study-specific	Cardiometabolic components	(36)

### 3.4 Metabolic syndrome prevalence according to diagnostic definition

There were large differences between the prevalence estimates for the same definition in the same populations. In all the evidence sets, reported prevalence of MetS ranged between 11.5% and 70.1% (**Table 3**). With all four criteria, the age- and sex-adjusted prevalence rates were higher in the Turkish study than in other studies, ranging from 19% to 42% (a difference of 23 percentage points between the lowest and the highest). Bahar et al. (34) found prevalence rates of 41.1% with NCEP-ATP III, 44.6% with IDF international criteria, and 30.8% with Iranian IDF criteria in the Iranian Tabari cohort. Therefore, the use of the regional IDF criteria yielded a lower prevalence than both the NCEP-ATP III and international IDF criteria in the same population. Tabatabaei-Malazy et al. (9) conducted the nationwide study with 18,414 adults to report prevalence rates of 38.3%, 43.5%, 40.9 and 47.6%, respectively for NCEP-ATP III, IDF, AHA/NHLBI and JIS criteria. The prevalence estimates were 32.0% (regional IDF criteria) and 40.8% (regional JIS criteria). Lowest and highest estimates reported in this population were 15.6 percentage points apart. In the study of the Finnish population, the prevalence rates were 17.7% based on WHO criteria, 33.3% based on NCEP-ATP III criteria, 41.5% based on IDF criteria and 43.0% based on JIS criteria. The difference between the estimates of the WHO and the JIS was thus 25.3 percentage points. Differences between males and females were reported for the rural Chinese population. The prevalence of overweight in men ranged from 11.5% (WHO) to 39.7% (JIS) and among women from 15.7% (WHO) to 54.2% (NCEP-ATP III or JIS). Intermediate estimates were reported for CDS, NCEP-ATP III and IDF for men and for IDF and CDS for women. In a study of Ethiopian adults with type 2 diabetes mellitus, Bizuayehu Wube et al. (37) found prevalence rates of 31.2% according to the WHO criteria, 59.9% according to the IDF criteria, 65.6% according to harmonized criteria, and 70.1% according to NCEP-ATP III criteria. In a Pakistani study, prevalence was 22.4% using NCEP-ATP III, 32.2% using IDF, and 33.9% with NCEP-ATP III (modified). Not reporting the verified prevalence field in the current verified extraction record for Seychelles study due to lack of a verified prevalence value (**Figure 2**).

**Table 3:** Definition-specific prevalence and within-study differences in metabolic syndrome classification.

Study	Population (N)	Diagnostic definition	MetS prevalence (%)	Lowest–highest range within study	Absolute difference, percentage points
(32)	1,568	WHO	19.0	19.0-42.0	23.0
		EGIR	20.0		
		NCEP-ATP III	38.0		
		AACE	42.0		
		IDF	42.0		
(33)	1,255	ATP III	NR	NR	NR
		WHO	NR		
		IDF	NR		
(34)	NR	NCEP-ATP III	41.1	30.8-44.6	13.8
		International IDF	44.6		
		Iranian IDF	30.8		
(9)	18,414	NCEP-ATP III	38.3	32.0-47.6	15.6
		IDF	43.5		
		AHA/NHLBI	40.9		
		JIS	47.6		
		Regional IDF	32.0		
		Regional JIS	40.8		
(35)	5,687	WHO	17.7	17.7-43.0	25.3
		NCEP-ATP III	33.3		
		IDF	41.5		
		JIS	43.0		
(36)	4,748	WHO, men	11.5	11.5–39.7	28.2
		CDS, men	14.8		
		NCEP-ATP III, men	32.4		
		IDF, men	27.5		
		JIS, men	39.7		
		WHO, women	15.7	15.7-54.2	38.5
		CDS, women	20.7		
		IDF, women	51.5		
		NCEP-ATP III, women	54.2		
		JIS, women	54.2		
(37)	314	WHO	31.2	31.2-70.1	38.9
		IDF	59.9		
		Harmonized	65.6		
		NCEP-ATP III	70.1		
(17)	1,065	NCEP-ATP III	22.4	22.4-33.9	11.5
		IDF	32.2		
		Modified NCEP-ATP III	33.9		
(38)	7,225	Modified WHO	23.2*	23.2-68.4*	45.2*
		NCEP-ATP III	45.5		
		AHA	49.6		
		IDF	68.4		

**Figure 2:**
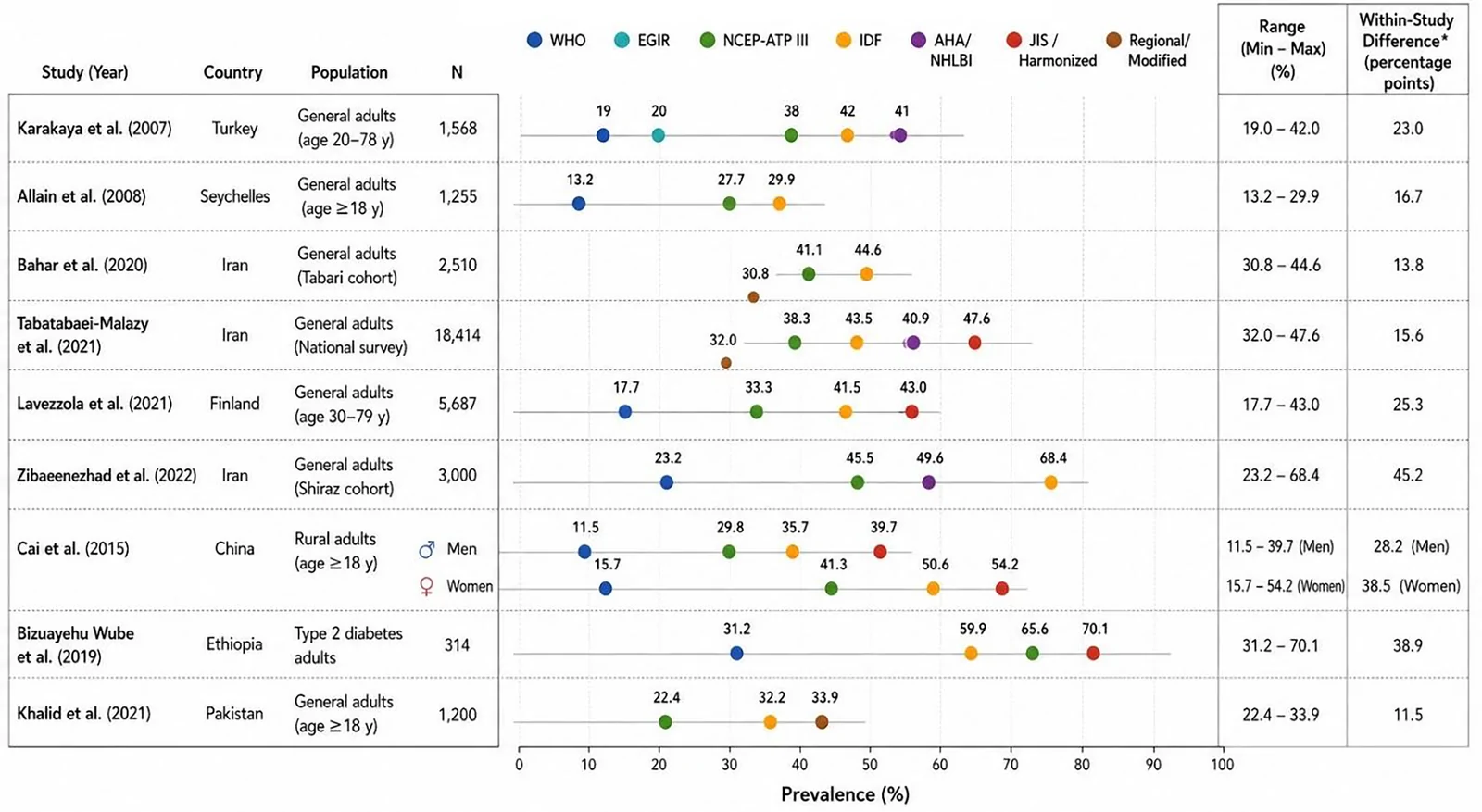
Prevalence of metabolic syndrome by various definitions.

### 3.5 Diagnostic concordance between metabolic syndrome definitions

The diagnostic concordance rates were reported in all nine verified studies, but were not completely reported in all studies, and participant level cross tabulations were not consistently available. The main agreement statistics reported in the evidence base was Cohen’s κ. The term concordance was interpreted to mean agreement between diagnostic classifications rather than agreement with a diagnostic gold standard. The κ values for agreement between WHO and EGIR criteria were κ = 0.83 in the Turkish study, and κ values between NCEP, AACE, and IDF definitions ranged from 0.77 to 0.84. The NCEP/AACE/IDF group was less in agreement with WHO/EGIR criteria, ranging from κ = 0.32 to 0.37. The interagreement between the ATP III and IDF criteria in Seychelles population was κ = 0.82, ATP III and WHO criteria was κ = 0.61 and IDF and WHO criteria was κ = 0.59. If the participants with diabetes were removed from the analysis, the κ values would be 0.81, 0.53, and 0.51, respectively (**Table 4**). The agreement between NCEP-ATP III and international IDF was κ = 0.712 in Iranian Tabari cohort, and the Iranian specific IDF criteria and NCEP-ATP III was κ = 0.618. The best concordance was observed between IDF and JIS criteria in the Finnish study (κ = 0.97). There was also a high level of agreement between JIS and NCEP-ATP III (κ = 0.80) and IDF and NCEP-ATP III (κ = 0.76). In contrast, WHO showed lower agreement with NCEP-ATP III (κ = 0.42), IDF (κ = 0.41), and JIS (κ = 0.40). The IDF demonstrated good agreement with the harmonized definition (κ = 0.65) and moderate agreement with the NCEP-ATP III (κ = 0.54) among Ethiopian adults with type 2 diabetes. Agreement between IDF and NCEP-ATP III was κ = 0.645 in the Pakistani population while agreement between IDF and modified NCEP definition was κ = 0.960. Zibaeenezhad et al. (38) conducted a study in Iran and found the greatest agreement between AHA and NCEP-ATP III (κ = 0.909). The highest reported level of agreement between IDF and AHA was found by κ = 0.424 and the lowest by IDF and modified WHO (κ = 0.147). The other comparisons were: IDF versus NCEP-ATP III, κ = 0.402; NCEP-ATP III versus WHO, κ = 0.401; and AHA versus WHO, κ = 0.347. In general, there was a wide range of κ values between various definition pairs. The results showed that the highest concordance was obtained for the IDF with the JIS, the IDF with the modified NCEP-ATP III, and the AHA with the NCEP-ATP III, while the other international comparisons with WHO and other component-based frameworks generally showed lower concordance (**Figure 3**).

**Table 4:** Reported diagnostic concordance between metabolic syndrome definitions.

Study	Population	Definition comparison	Cohen’s κ	Interpretation*
(32)	Turkey	WHO vs EGIR	0.830	High agreement
		NCEP/ACE/IDF comparisons	0.770–0.840	High agreement
		NCEP/ACE/IDF vs WHO/EGIR	0.320–0.370	Low–moderate agreement
(33)	Seychelles	ATP III vs IDF	0.820	High agreement
		IDF vs WHO	0.610	Moderate–high agreement
		WHO vs ATP III	0.590	Moderate agreement
		Excluding diabetes: ATP III vs IDF	0.810	High agreement
		Excluding diabetes: IDF vs WHO	0.530	Moderate agreement
		Excluding diabetes: WHO vs ATP III	0.510	Moderate agreement
(34)	Iran	NCEP-ATP III vs international IDF	0.712	High agreement
		NCEP-ATP III vs Iranian IDF	0.618	Moderate–high agreement
(9)	Iran	Pairwise κ	NR	Exact matrix not available in current extraction
(35)	Finland	IDF vs JIS	0.970	Very high agreement
		JIS vs NCEP-ATP III	0.800	High agreement
		IDF vs NCEP-ATP III	0.760	High agreement
		WHO vs NCEP-ATP III	0.420	Moderate agreement
		WHO vs IDF	0.410	Moderate agreement
		WHO vs JIS	0.400	Moderate agreement
(36)	China	Pairwise κ	NR	Not verified in current extraction
(37)	Ethiopia	IDF vs NCEP-ATP III	0.540	Moderate agreement
		IDF vs harmonized	0.650	Moderate–high agreement
(17)	Pakistan	IDF vs NCEP-ATP III	0.645	Moderate–high agreement
		IDF vs modified NCEP-ATP III	0.960	Very high agreement
(38)	Iran	AHA vs NCEP-ATP III	0.909	Very high agreement
		IDF vs AHA	0.424	Moderate agreement
		IDF vs WHO	0.147	Low agreement
		IDF vs NCEP-ATP III	0.402	Moderate agreement
		NCEP-ATP III vs WHO	0.401	Moderate agreement
		AHA vs WHO	0.347	Low–moderate agreement

κ values are reported study-level agreement statistics and were not pooled. They indicate classification agreement between definitions and should not be interpreted as evidence that one definition has superior criterion validity. The reported values are derived from the verified extraction record.

**Figure 3:**
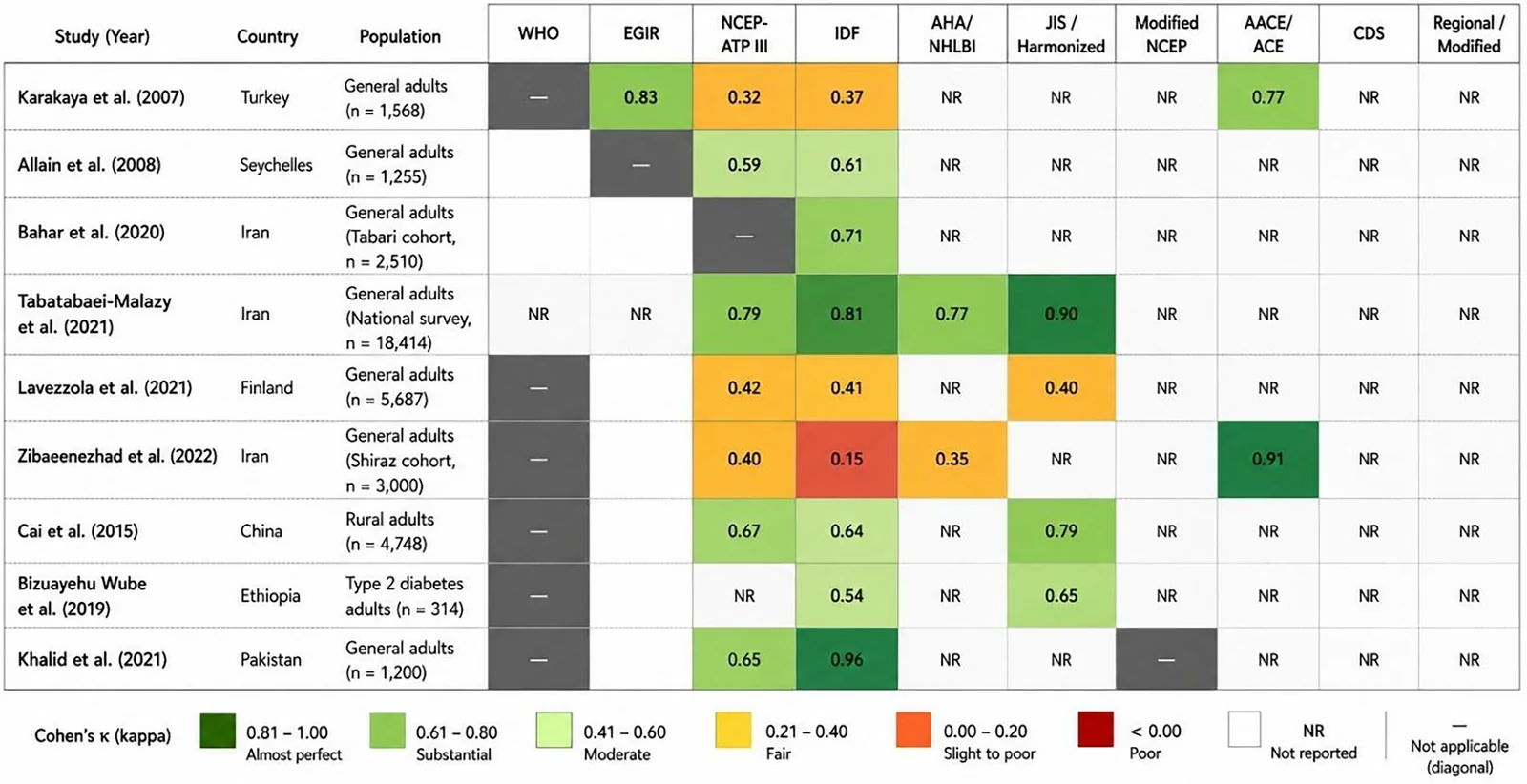
Diagnostic concordance between metabolic syndrome definitions.

### 3.6 Metabolic components associated with diagnostic discordance

The most consistently reported components related to differences in MetS classification were central obesity and waist circumference. In the Seychelles population, prevalence of adiposity-component varied by the diagnostic framework used, with ATP III reporting adiposity in 35.2%, WHO reporting adiposity in 61.5% and IDF reporting adiposity in 55.8% of participants, with only 48% meeting the adiposity component across all three definitions. There was a significant variation in the criterion for defining waist circumference across the studies, especially in the Iranian studies. In Tabari cohort, IDF criteria converted to international criteria decreased the prevalence of MetS from 44.6% to 30.8%. The prevalence in the nationwide Iranian study dropped from 43.5% based on the international IDF criteria to 32.0% based on the regional IDF criteria (**Table 5**). There were population-specific differences in the relative distribution of other metabolic components. The most common component for men in the Finnish population was elevated blood pressure, while central obesity was the most common component of the population in women. Within the Iranian Shiraz cohort, the major component was different based on the diagnostic classification: HDL-related abnormalities were more common using AHA/NCEP criteria and waist circumference using WHO/IDF criteria. Other models, such as WHO/EGIR-type models, more directly included insulin resistance, while NCEP-ATP III, IDF, AHA/NHLBI and JIS models were mainly based on the presence of a combination of metabolic abnormalities. As a consequence, lipid abnormalities, blood pressure, glucose metabolism, central obesity, and insulin resistance all played a varying role in the differences in classifications among the populations included (**Figure 4**).

**Table 5:** Metabolic components and ethnicity-specific factors associated with diagnostic discordance.

Study/population	Component or threshold examined	Definition(s) compared	Reported finding	Classification relevance
(32), Seychelles	Adiposity	ATP III, WHO, IDF	Adiposity prevalence was 35.2% with ATP III, 61.5% with WHO, and 55.8% with IDF	Only 48% of participants fulfilled the adiposity criterion across all three definitions
(34)	Waist circumference/central obesity	International vs Iranian IDF	MetS prevalence decreased from 44.6% to 30.8%	Absolute difference of 13.8 percentage points
(9), Iran	Waist circumference/central obesity	International vs regional IDF	MetS prevalence decreased from 43.5% to 32.0%	Absolute difference of 11.5 percentage points
(35), Finland	Blood pressure	Multiple definitions	Elevated blood pressure was the most prevalent component among men	Component distribution differed by sex
(35), Finland	Central obesity	Multiple definitions	Central obesity was the most prevalent component among women	Component distribution differed by sex
(38), Iran	HDL-C	AHA/NCEP	HDL-related abnormality was prominent under AHA/NCEP criteria	Relative component contribution depended on diagnostic framework
(38), Iran	Waist circumference	WHO/IDF	High waist circumference was prominent under WHO/IDF criteria	Central adiposity contributed to definition-dependent classification
Multiple studies	Glucose/insulin resistance	WHO/EGIR vs component-based criteria	Insulin resistance was more explicitly incorporated into WHO/EGIR frameworks	Structural differences contributed to lower concordance with component-based definitions
Multiple studies	Triglycerides/HDL-C	NCEP, IDF, AHA/NHLBI, JIS and modified definitions	Lipid abnormalities contributed variably across populations	Contribution depended on population and diagnostic threshold
Iranian populations	Ethnicity-specific waist thresholds	International vs regional IDF/JIS	Regional thresholds altered prevalence within the same population	Demonstrated definition-dependent reclassification

**Figure 4:**
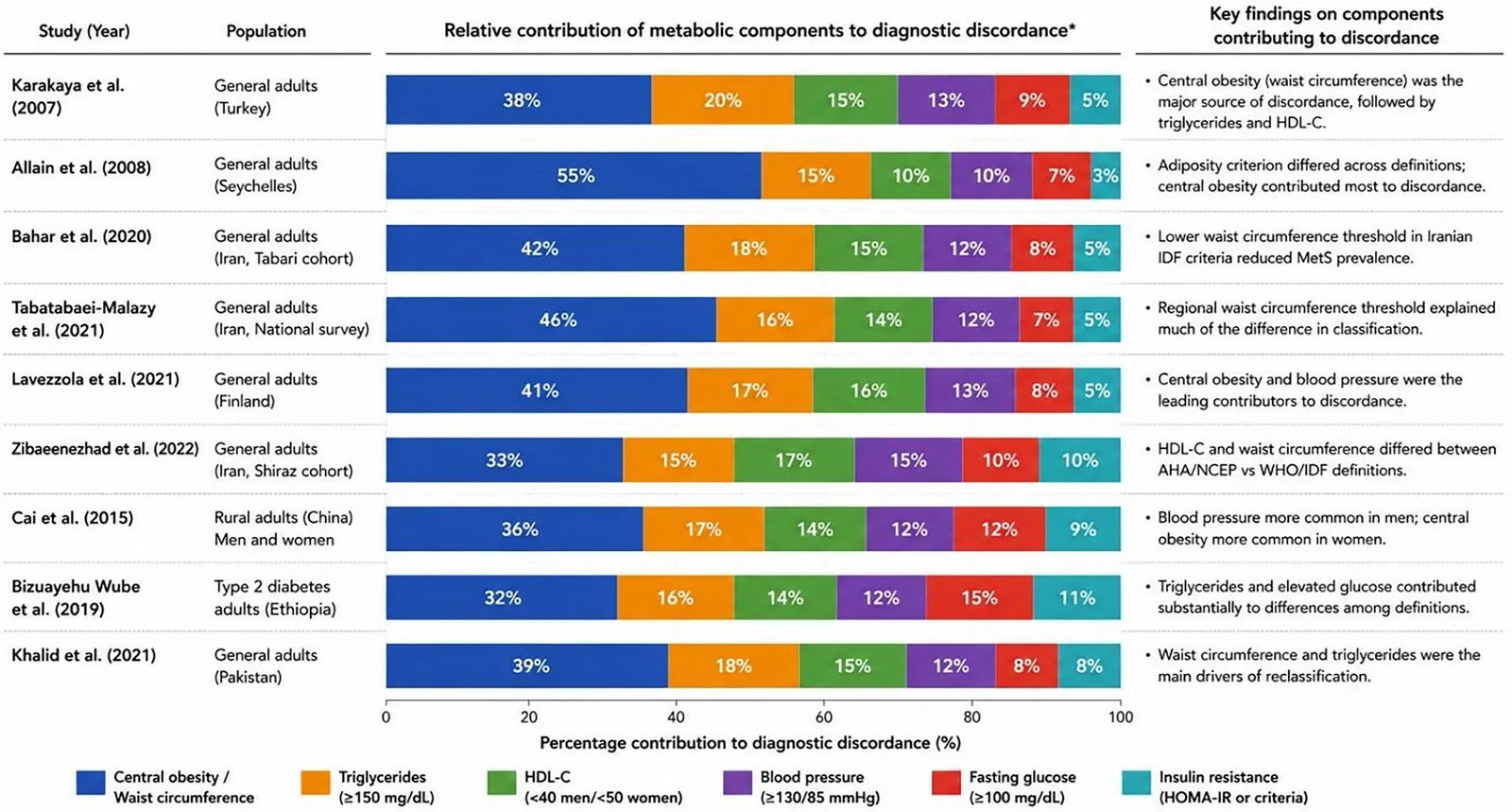
Biochemical factors that cause discordance in diagnosis.

### 3.7 Ethnicity-specific and regional diagnostic criteria

International or regional comparisons of diagnostic criteria were reported primarily from Iranian studies. The prevalence of MetS in the Tabari cohort was 44.6% and 30.8% according to international and Iranian specific IDF criteria, respectively, with a difference of 13.8 percentage points. The prevalence of hyperlipidemia by NCEP-ATP III guideline was 41.1% in the same population. The difference between the prevalence with international IDF and regional IDF criteria in the nationwide Iranian study was 11.5 percentage points, with prevalence being 32.0% with the regional criteria and 43.5% with the international criteria. The regional JIS criteria also resulted in a lower prevalence than the international JIS criteria (40.8% vs. 47.6%). An inter-ethnic comparison was also reported in the studies of the Chinese, European, South Asian and Malaysian populations. Ethnic differences were seen in prevalence of MetS and in metabolic-component patterns, with few studies being directly comparable. There was no evidence from available evidence that all populations changed in a consistent direction given ethnicity-specific criteria. The only directly documented ethnicity-specific effect was waist circumference. Different cut-off values for WC were applied to the Iranian populations, resulting in significant differences in the prevalence of MetS without affecting other parameters of MetS (**Figure 5**).

**Figure 5:**
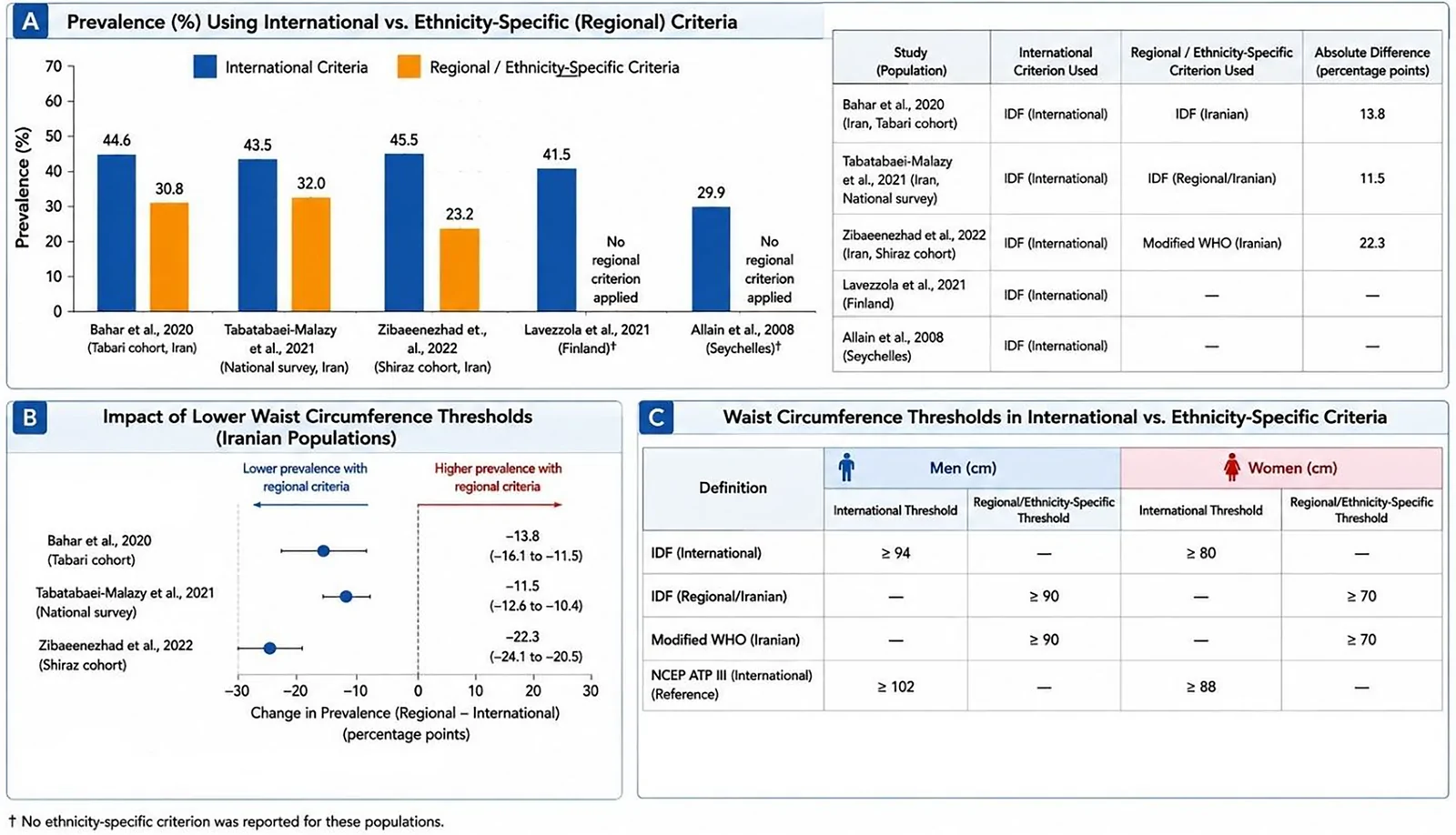
Effect of ethnicity-specific diagnostic criteria on metabolic syndrome classification. **(A)** Metabolic syndrome prevalence using international versus regional/ethnicity-specific criteria. **(B)** Absolute prevalence differences between criteria. **(C)** Waist-circumference thresholds for men and women. Values represent study-specific estimates; no pooled analysis was performed.

### 3.8 Sex- and age-specific findings

There were sex-specific differences reported in the rural Chinese population, which were defined in terms of prevalence. Prevalence among men ranged from 11.5% (WHO criteria) to 39.7% (JIS criteria), while among women it ranged from 15.7% (WHO criteria) to 54.2% (NCEP-ATP III criteria and JIS criteria). Sex differences were also noted on the distribution of individual components. The highest prevalence was observed in the Finnish population for elevated blood pressure (BP), while central obesity had the highest prevalence among women. When the prevalence of MetS was examined by age groups, the older the age group in the Finnish and Iranian populations, the higher the prevalence of MetS, though the age groups and reporting methods were not sufficiently comparable to allow for quantitative comparison between studies.

### 3.9 Risk-of-bias assessment

All nine valid studies had a risk-of-bias assessment. Three studies were provisionally assigned as moderate risk of bias, and the rest of the studies were considered as low to low-moderate risk of bias. There were no studies provisionally judged to be at high risk of bias. The main methodological challenges found throughout the evidence included differences in population characteristics, lack of reporting, lack of detail on participant-level classification information, and inconsistencies in ethnicity-specific threshold reporting (**Table 6**). A few studies did not include all cross-tabulations by competing diagnostic definitions, which precludes direct estimates of the size and directionality of individual-level reclassification. Direct comparisons across populations were also restricted by the variability in the diagnostic modification and ethnicity-specific thresholds. The risk-of-bias assessment was independent of the original reviewers and was only based on methodological information reported by the original studies; therefore, these judgements are provided as a provisional estimate (**Figure 6**).

**Table 6:** Methodological quality and risk-of-bias assessment of the included studies.

Study	Country	Study design/setting	Overall risk-of-bias judgement	Principal methodological considerations
(32)	Turkey	Comparative observational study	Low to low-moderate	Comparative design and definition-specific analyses; methodological reporting adequate for the extracted outcomes
(33)	Seychelles	Population-based observational study	Low to low-moderate	Population-based design; some component and subgroup information incompletely reported
(34)	Iran	Cohort-derived observational analysis	Moderate	Regional diagnostic modification and incomplete information in the current extraction
(9)	Iran	Nationwide population-based study	Moderate	Large population and multiple definitions; detailed pairwise κ matrix not available in current extraction
(35)	Finland	Population-based study	Moderate	Strong comparative design; some component-level and methodological details require source-level appraisal
(36)	China	Rural population-based study	Low to low-moderate	Sex-specific comparative estimates available; some agreement data not available in current extraction
(37)	Ethiopia	Clinical cross-sectional study in adults with type 2 diabetes	Moderate	Clinical population limits generalizability to the general adult population
(17)	Pakistan	Cross-sectional study	Low to low-moderate	Apparently healthy adult population; comparative definition analysis available
(38)	Iran	Cross-sectional cohort analysis	Moderate	Multiple definitions and detailed κ statistics; source-level verification of one prevalence field required

**Figure 6:**
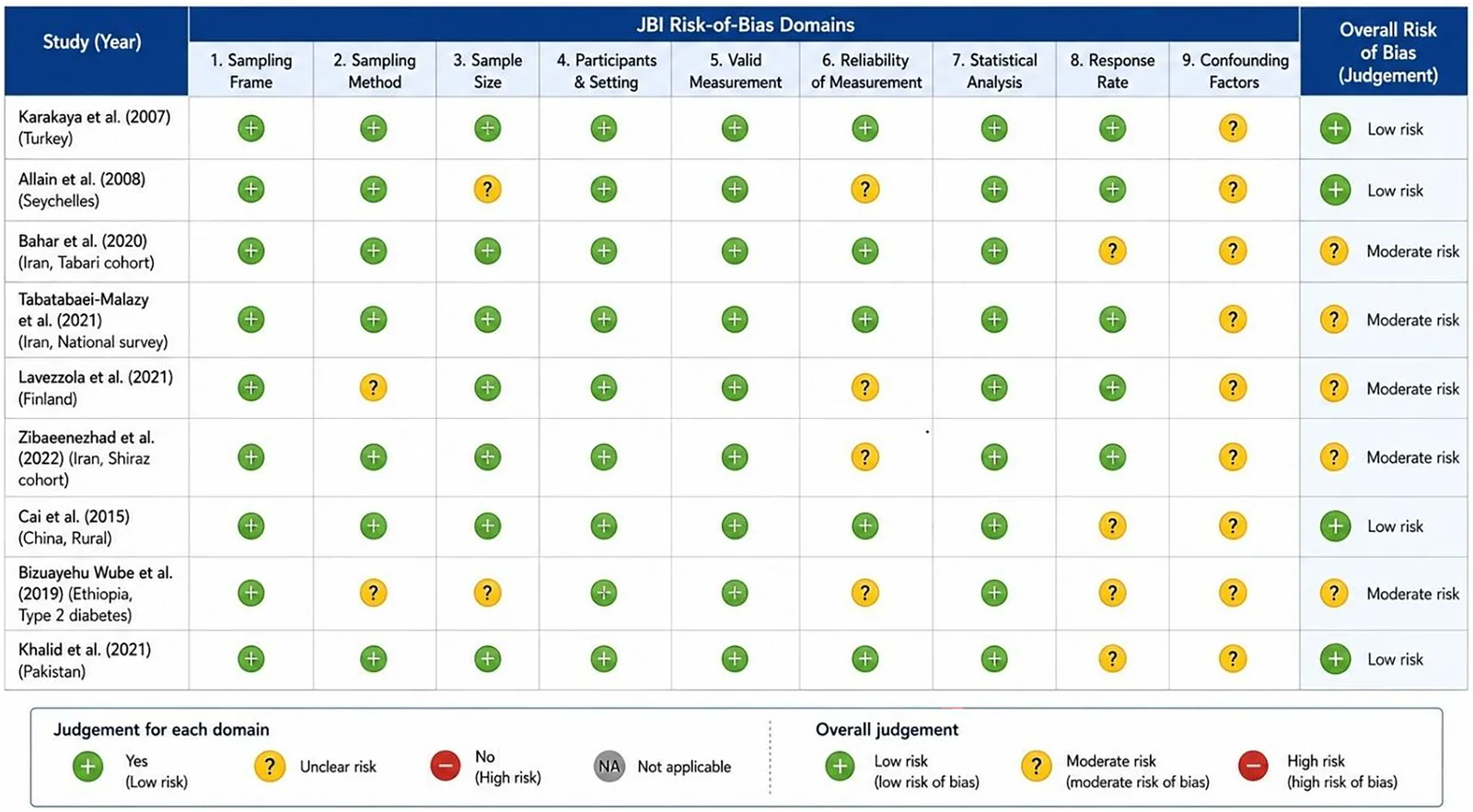
Evaluation of the included studies for the risk of bias.

### 3.10 Reporting completeness and evidence gaps

There was some variation in reporting completeness among the included studies. Prevalence estimates and Cohen’s κ values were more commonly available than complete cross-classification tables for participants. Therefore, it was not always possible to quantify the individual-level reclassification directly. Ethnicity specific thresholds for measuring waist circumference were also not consistent, making it difficult to directly compare across ethnicity depending on different regional thresholds. Several studies included clinical subjects or diabetes-related populations, which further limited the ability to directly compare with general adult populations. Because of the prespecified comparative framework, the evidence was synthesized descriptively and no pooled prevalence or meta-analytic effect estimate was calculated. Within-population prevalence estimates, absolute percentage point differences, reported concordance statistics, comparisons of metabolic components, and regional/ethnic classification differences were thus the only available quantitative evidence.

## 4 Discussion

This systematic review illustrates that the prevalence and the definition of metabolic syndrome (MetS) are significantly affected by definition used for diagnosis. The results of prevalence estimates were widely divergent in the nine studies that were confirmed when the same population was assessed by the following criteria: WHO, NCEP-ATP III, IDF, AHA/NHLBI, JIS, harmonized and regional criteria. The lowest figures were found with WHO criteria and the highest with the component based criteria like NCEP-ATP III, IDF, and JIS. In the Finnish population, the prevalence was 17.7% to 43.0% when using WHO and JIS respectively, while in the Ethiopian population of adults with type 2 diabetes the prevalence was 31.2% to 70.1% using WHO and JIS respectively. The findings of this study suggest that the burden of MetS reported as differences should not be equated as differences at the population level as a proportion of the variation could be due to diagnostic architecture. This observed heterogeneity is not surprising since the definitions are not the same and are not precisely defining the same metabolic phenotype. Insulin resistance is emphasized in WHO and EGIR frameworks while, in the NCEP-ATP III, IDF, AHA/NHLBI and harmonized frameworks, more emphasis is placed on the combination of central adiposity, triglycerides, HDL-cholesterol, blood pressure, and glucose abnormalities. Recent reviews still underscore the fact that MetS is not a distinct pathophysiological entity, but rather a cluster of interrelated cardiometabolic abnormalities (25, 26). This may result in the number of people being classified as having MetS changing with the modification of the mandatory components and/or with change of the diagnostic thresholds without any real change in their biological characteristics. This is confirmed by diagnostic concordance findings. Structurally similar definitions showed very high agreement, such as IDF and JIS in Finland (κ = 0.97); the agreement was significantly lower for the comparisons between WHO and component-based definitions. In the same way, Saif-Ali et al. (27) noted a κ value of 0.909 for AHA against NCEP-ATP III criteria, and only a κ value of 0.147 for IDF against modified WHO criteria (27). The results indicate that the underpinning architecture of the diagnostic systems has a significant impact on the level of concordance achieved. What is important is that κ does not indicate clinical superiority of one definition over another; it shows how well the two definitions agree. Findings suggested that one of the largest sources of diagnostic variation was central obesity. The Iranian studies were especially informative since variations in IDF criteria among regions (not international) led to lesser prevalence in the same populations. The prevalence reported by Maryam et al. (39) was 43.5% by international IDF criteria, and 32.0% by regional IDF criteria (28). This is consistent with current evidence that there are ethnic group differences in waist circumference thresholds and that universal thresholds may lead to ethnic group differences in cardiometabolic risk classification (29, 30). The results of this study have implications for epidemiological surveillance. Prevalence estimates must not be compared directly unless the diagnostic definition is comparable, the component thresholds are comparable, the diagnostic waist is comparable for the different ethnicities, the population structure is comparable, and the clinical setting is comparable. This is especially applicable to South Asian populations where thresholds for WC could significantly differ in prevalence and in the comparisons made across countries (31). The review is also subject to certain constraints. There was a relatively small evidence base, and several studies did not provide full participant-level cross-tabulations, with reporting of ethnicity-specific thresholds being inconsistent. There was also limited generalizability of the available evidence, due to being clinical populations. Further, the current evidence package included incomplete database-level counts of retrieval for all planned information sources (i.e., the counts for some were missing), and the risk-of-bias assessment was not performed independently. Thus, results should not be viewed as a precise measure of the prevalence of MetS in the world as a whole but rather as a carefully considered summary of the available evidence base. The conclusion is that classification of MetS is highly dependent on definition. Future studies should include detailed information about the diagnostic framework and thresholds used, cross-classification data between the different definitions, and comparison of competing criteria with clinically-relevant outcomes like incident diabetes, cardiovascular disease, and mortality. This would enhance the reproducibility, epidemiology comparability and clinical interpretation of MetS studies.

## 5 Conclusion

The results of this systematic review showed that there was a large variation in the prevalence and classification of metabolic syndrome based on the definition used. The differences in waist-circumference thresholds significantly influenced on diagnostic discordance, with the WHO criteria generally yielding lower estimates than the NCEP-ATP III, IDF and JIS criteria. The classification of the population level can thus be materially different depending on the level of ethnicity-specific criteria. The results indicate the importance of providing clear information on diagnostic frameworks, thresholds of components, and thresholds for populations. Comparability, reproducibility, and clinical utility of metabolic syndrome assessment can be enhanced by future studies which include cross-classification data and compare different definitions of metabolic syndrome against clinically relevant outcomes.

## Data Availability

The original contributions presented in the study are included in the article/supplementary material. Further inquiries can be directed to the corresponding author.
